# Dietary Supplement Use among Australian Adults: Findings from the 2011–2012 National Nutrition and Physical Activity Survey

**DOI:** 10.3390/nu9111248

**Published:** 2017-11-14

**Authors:** Alissa J. Burnett, Katherine M. Livingstone, Julie L. Woods, Sarah A. McNaughton

**Affiliations:** School of Exercise and Nutrition Sciences, Institute for Physical Activity and Nutrition, Deakin University, 221 Burwood Highway, Burwood, VIC 3125, Australia; k.livingstone@deakin.edu.au (K.M.L.); j.woods@deakin.edu.au (J.L.W.); sarah.mcnaughton@deakin.edu.au (S.A.M.)

**Keywords:** supplements, dietary intake, lifestyle

## Abstract

(1) Background: Supplement use is prevalent worldwide; however, there are limited studies examining the characteristics of people who take supplements in Australia. This study aimed to investigate the demographics, lifestyle habits and health status of supplement users; (2) Methods: Adults aged >19 years (*n* = 4895) were included from the 2011–2012 National Nutrition and Physical Activity Survey (NNPAS). A supplement user was defined as anyone who took one or more supplements on either of two 24-h dietary recalls. Poisson regression was used to estimate the prevalence ratio (PR) of supplement use, according to demographics, lifestyle characteristics and health status of participants; (3) Results: Supplement use was reported by 47% of women and 34% of men, and supplement use was higher among older age groups, among those with higher education levels and from areas reflecting the least socioeconomic disadvantaged. An association was found between blood pressure and supplement use; (4) Conclusions: A substantial proportion of Australians take supplements. Further investigation into the social, psychological and economic determinants that motivate the use of supplements is required, to ensure appropriate use of supplements among Australian adults.

## 1. Introduction

The use of supplements, which may be defined as multi-vitamins, single vitamins, single minerals, herbal supplements, oil supplements and any other dietary supplementation [1] varies among populations. However, it is highest in countries such as the United States, United Kingdom and Denmark [2], where supplement use is between 35 and 60% of adults [1,2,3,4,5]. On a per capita basis, Australians are some of the world’s largest consumers of dietary supplements, with vitamin and mineral sales totaling AUD $646 million in 2013 [3].

The Australian Dietary Guidelines recommend that individuals, with the exception of pregnant women, only take supplements if they are eliminating a food group from their diet [6]. However, many people continue to take supplements. Supplement users may differ from non-users in regard to a range of characteristics. Previous research suggests that supplement users are more likely to be female, older and have a higher educational attainment than non-supplement users [1,2,3,4,5,7]. Supplement use is also higher in people who adopt healthier lifestyle behaviours, such as being more physically active, not smoking and consuming more fruit and vegetables and less alcohol [1,8,9]. Several studies show that less healthy diets are common among supplement non- users, including greater consumption of diets high in fat, low in fibre or low in fruit [8,9].

Just as supplement users have been shown to maintain healthier lifestyle behaviours, individuals who take dietary supplements are more likely to have a better health status [2,8,10] including better self-rated health and fewer cardiovascular risk factors, such as high blood pressure, than non-supplement users.

While supplement users may have more optimal dietary intakes compared to non-supplement users [11], many supplement users have also been found to be exceed the upper recommended intake limit for some vitamins and minerals [11], which is defined as the highest level of individual daily intake that does not pose a threat to health [11]. For example, the Multiethnic Cohort study in the United States reported that, of people who take supplements, 50% of men and 40% of women consumed more than the upper limit for niacin and folate [11].

Studies to date that have characterized supplement users, have lacked detailed dietary records and supplement information, and there has been a paucity of nationally-representative data on supplement use in the Australian population. Given the high prevalence of consumption and the limited understanding of the characteristics of these consumers, this study aimed to examine supplement use within the Australian population. Specifically, the present study aimed to explore the sociodemographic, lifestyle and health status characteristics of supplement users; the dietary intakes of supplement users and the most common types of supplements used among Australian adults.

## 2. Methods

### 2.1. Study Design and Participants

This study was a secondary analysis of data collected from adults included in the National Nutrition and Physical Activity Survey 2011–2012 (NNPAS), a component of the Australian Health Survey [12]. The NNPAS collected information from a random sample of private dwellings in both urban and rural areas across all Australia states and territories. A total of 14,363 private dwellings were selected, of which 9519 (77.0%; *n* = 12,153 individuals, 9341 adults >19 years) were fully or adequately responding households to the first interview. Participants completed a household survey, anthropometric measures and dietary intakes were estimated using two 24-h dietary recalls. For the present study, participants were excluded if they were pregnant or lactating, completed only one 24-h dietary recall, or had missing data on outcomes and covariates. The final sample for analysis was 4895. Use of the NNPAS 2011–2012 was approved by the Australian Bureau of Statistics [13] and ethics approval was provided by The Census and Statistics Act 1905 [12].

### 2.2. Dietary Intake

A 24-h dietary recall collected detailed information on all foods and beverages consumed in a 24-h period, from midnight to midnight. Where possible, the participants were asked to undertake a second 24-h dietary recall, at least eight days after their first recall on a different day of the week. The 24-h dietary recall method was based on the Automated Multiple-Pass Method, developed by The United States Department of Agriculture (USDA), and adapted for use in Australia [14]. The Automated Multiple-Pass Method is divided into five phases: quick list, forgotten foods, time and occasion, detail cycle, and final probe. The information collected included time of consumption, the name of the eating occasion (e.g., lunch), the amount eaten and detailed food descriptions to allow for accurate food coding. Energy (kJ) and nutrient intakes (fat, protein, carbohydrates and fibre (g) and calcium, magnesium, iron, zinc, iodine, selenium, vitamin C, vitamin E (mg), B12 (µg), long chain omega 3 fatty acids, and folate (natural) and folic acid (µg)) were estimated from the 24-h recalls, using the Australian Supplement and Nutrient Database 2011–2013 [15].

Overall, diet quality was assessed using the dietary guideline index (DGI-2013), which is described in detail elsewhere [16,17,18]. It is calculated using 13 components, with seven of the 13 components assessing the adequacy of the diet (e.g., food variety, vegetables, fruits, cereals, dairy and alternatives, lean meat and alternatives, and fluid intake), and the remaining six components assessing the moderation of dietary intake (e.g., discretionary foods, saturated fat, salt, added sugar and alcohol, and moderate intakes of unsaturated fat). Each component is scored from 0 to 10, to reflect the level of compliance for meeting the Australian dietary guidelines and scores on each component were summed to give the total score. A higher score reflects a better diet quality. This was then categorised into two groups, based on the median (high score or low score).

### 2.3. Dietary Supplement Use

At the end of the dietary recall, participants were also asked “were dietary supplements, such as vitamins and minerals consumed, and how much?”. Participants were asked to report their supplement usage, using the Australian Register of Therapeutic Goods (ARTG) identification number on the supplement container. These were then matched to a list provided by the Therapeutic Goods Administration of over 10,000 dietary supplements registered for sale in Australia. For the purposes of this study, a supplement user was defined as anyone who took one or more supplements during either of the two 24-h dietary recalls.

### 2.4. Socio-Demographic Measures

Information on respondents’ socio-demographics and other characteristics was collected in the household survey. Age (years) was grouped into four categories—19–30, 31–50, 51–70 and 71–85 years—consistent with age groups used in the Australian Nutrient Reference Values [19]. Education was assessed by asking respondents to provide details of their highest education levels, both school and non-school, and categorised as secondary school or less, certificate or diploma, bachelor degree and postgraduate degree. Area-level disadvantage was assessed using the Australian Bureau of Statistics (ABS) Socio-Economic Indexes for Areas (SEIFA), and specifically, the Index of Relative Socio-economic Disadvantage [20], which was compiled from variables such as income, educational attainment, unemployment, and dwellings without motor vehicles [20]. For the present study, this was categorised into quintiles, with the first quintile representing the most disadvantaged group.

### 2.5. Lifestyle Characteristics

Information on health behaviours was also collected in the household survey. Frequency and duration of moderate and vigorous physical activity in the last week were assessed in the NNPAS, using questions from the Active Australia Survey [21]. Physical activity was measured in relation to Australia’s Physical Activity and Sedentary Behaviour Guidelines, which recommend at least 30 min of moderate intensity physical activity on most, preferably all, days [22]. Physical activity included walking for fitness, recreation or sport for at least 10 min, walking continuously to get from place to place for at least 10 min, moderate physical activity/exercise (apart from walking) and vigorous physical activity/exercise. A binary variable was used to estimate whether participants met recommendations of 150 min of physical activity over five or more sessions per week. Sedentary behaviour (min/day) was defined as time spent sitting or lying down for various activities in the last week; participants were asked to record the number of minutes they had spent sitting or lying down on a usual day. Smoking was categorised as current smoker, ex-smoker and never smoked.

Alcohol was assessed by asking respondents to report the number of drinks, alcohol type, size of drinks and brand name of drink they had consumed [23]. The reported quantities were then converted into millilitres of alcohol present in the reported drinks. For the purpose of this study, alcohol intake was then categorised into three categories; no alcohol consumed, one standard drink or less consumed and more than one standard drink consumed. A standard drink was defined as 10 grams of alcohol [6].

Usual fruit and vegetable intake was measured by asking respondents to report the number of serves of fruit and vegetables they ate each day [24]. This was then categorised into whether the participant met the guidelines or not (five serves of vegetables and two serves of fruit per day) [6].

### 2.6. Health Status Measures

Self-reported health was measured by asking the participants to report their self-perceived health as excellent, very good, good, fair and poor [25]. Fair and poor were grouped together due to a low number of responses. Weight (kg) was measured using digital scales. This was measured during the interview and was voluntary. Body mass index (BMI; kg/m^2^) was calculated using Quetelet’s metric BMI, which is calculated as weight (kg) divided by height (m^2^). This was then categorised into groups: underweight, normal, overweight and obese. Height was measured using a stadiometer. Waist circumference (cm) was measured using a metal tape measure, at the approximate midpoint between the lower margin of the last palpable rib and the top of the iliac crest [26]. This was measured during the interview and was voluntary. Waist circumference was categorised into three groups: no risk, increased risk and substantially increased risk. Blood pressure was measured by the interviewer using an automated blood pressure monitor and was voluntary. Two measurements were taken while participants were sitting down. If there was a significant difference between the two readings (greater than 10 mmHg), a third reading was taken. If there was no significant difference between the two readings, the second reading was recorded [27]. These measurements were then categorised into two groups (<140/90 and >140/90 mmHg). Chronic disease was measured by asking the participants whether they had been diagnosed with either diabetes, heart disease or kidney disease. This was then categorised into two groups (present or absent).

### 2.7. Statistical Analysis

Data analysis was conducted using Stata 14.2 (Stata Corp., College Station, TX, USA) [28]. Descriptive statistics (frequencies, mean and standard error) were used to describe variables and characteristics of supplement users versus non-users were compared using independent *t*-tests (for continuous variables) and chi square analyses (for categorical variables). Dietary intakes were log transformed for nutrients that were not normally distributed (total zinc, food only vitamin C, total vitamin C, total vitamin E, food only folic acid, total folic acid, food only B12, total B12, food only long chain omega 3 fatty acids and total long chain omega 3 fatty acids). Poisson regression analyses were used to estimate the prevalence ratio (PR) of supplement use (dependent variable), according to demographics, lifestyle characteristics and health status of participants (independent variables). Poisson regression was used to account for robust error variance. Analyses for socio-demographic measures, lifestyle characteristics and self-related health were adjusted for age (continuous) and sex, while health-related characteristics were adjusted for age, sex, education, area level disadvantage, smoking, fruit and vegetable consumption and DGI. Sampling weights were applied to take account of the complex sampling design. For categorical variables, a post hoc analysis was conducted (*F*-test) to test the overall effect of the variable on the dependent variable. Statistical significance was set at *p* < 0.05 for the analysis.

## 3. Results

Overall, supplement use was reported by 34% of men and 47% of women (Table 1). Supplement use was highest among women, among those aged 71–85 years, those with highest levels of education, those living in areas with the least socio-economic disadvantage, those who met physical activity guidelines and those who met fruit and vegetable guidelines (*p* < 0.05). After adjustment for age (Table 2), the prevalence of supplement use was higher in women and when adjusted for sex, the prevalence of supplement use was higher in older adults (71–85 years). When adjusted for sex and age, the prevalence of supplement use was higher in those in the least area level disadvantaged group, those with higher educational attainment, those who met the guidelines for physical activity (PR 0.79; 95% CI 0.71, 0.88; *p* < 0.001) and fruit and vegetable intakes (PR 0.81; 95% CI 0.68, 0.97; *p* < 0.05), and those with higher DGI scores (PR 1.32; 95% CI 1.20, 1.44; *p* < 0.001). An association was found between being an ex-smoker (PR 1.56; 95% CI 1.31, 1.86; *p* < 0.001) or having never smoked (PR 1.46; 95% CI 1.25, 1.70; *p* < 0.001) and supplement use, when compared with current smokers. No association was found between alcohol intake or sedentary behaviour and supplement use. An inverse association (Table 3) was found between being hypertensive (PR 0.87; 95% CI 0.77, 0.99; *p* = 0.031) and waist circumference (PR 0.89; 95% CI 0.79, 1.01; *p* = 0.007) and supplement use. No association was found between BMI, self-assessed health, chronic disease and supplement use.

The most commonly used supplements were single vitamins (19%), herbal supplements (16%), and multivitamins (16%) (Table 4). The most commonly used supplements used varied by age group, with folic acid being most commonly consumed by the 31–50 year age group, while lipids (e.g., fish oil supplements, fish oil supplements with added nutrients, fish liver oil supplements, evening primrose oil supplements and other lipid supplements grouped together) were most commonly consumed by the 51–70 year age group. Females consumed a higher proportion of multivitamins (62%), single minerals (67%), single vitamins (60%), herbal supplements (63%), iron (62%) and folic acid (59%) than males.

When comparing nutrient intakes from food only, supplement users were found to have higher intakes of fibre and most vitamins and minerals (except, zinc and vitamin B12) compared to non-supplement users, although differences were small in many cases (Table 5). Intakes of all vitamins and minerals were higher when the contribution from supplements was added into overall intakes. Intakes of folic acid from food alone were lower among supplements users, but the inclusion of supplement intakes (in the calculation of total intake from food and supplements) reversed the differences. Supplement users were found to be reaching the upper range of the recommended dietary intake (RDI) for magnesium and exceeding the RDI for zinc, vitamin C, vitamin E and B12, when total intakes were considered.

## 4. Discussion

A significant proportion of the Australian population reported supplement use (34% men and 47% women), with supplements users more likely to be female, older, more highly educated and to exhibit healthier lifestyle behaviours and have better health status than supplement non-users. Nutritional supplements are considered complementary medicines within Australia, and undergo less regulation than higher risk products, such as medicines [30]. Therefore, less emphasis is placed on assessing the evidence of the claims being made by the products; these marketing claims may persuade people to consume more dietary supplements [30,31].

Our findings, in relation to the sociodemographic characteristics of supplement users, are consistent with the literature [1,2,4,5,7,9,31,32,33]. Many previous studies have found that supplement users are more likely to be female, older in age and more highly educated [1,2,4,5,7,9,31,32,33]. This is reflected within the current study, as the largest percentage of supplement users were older in age. Females have been shown to be more health conscious and therefore may take more dietary supplements to prevent illness [34].

Previous studies have also found that people of a lower socio-economic position are less likely to use supplements, which is reflected within the current study [9,31]. Previous studies have shown that people with a higher socio-economic position are more likely to be more health conscious, which may motivate them to take more dietary supplements [5].

Previous studies suggest that supplement users are more likely to engage in a range of health behaviors and are more likely to meet recommendations for physical activity and fruit and vegetable consumption. Previous research has shown that supplement users are more likely to be physically active [1,2,4,9,35]. The current study found a relationship between supplement use and meeting the physical activity guidelines; however, there was no significant difference found between supplement users and supplement non-users, with regard to sedentary behaviour. It is consistently shown that supplement use is associated with health conscious individuals; however, the lack of association may be due to the inability to modify sedentary behaviour, as much of it occurs in the workplace [36]. Meeting the guidelines for fruit and vegetable consumption was positively associated with dietary supplement use in the present study. This is consistent with previous studies, which found an association between higher consumption of fruit and vegetables and dietary supplement use [2,9,37]. This introduces the question of the need for additional nutritional supplementation in those who already obtain nutrients from a healthy diet, as supplement users’ dietary intakes were generally better and met the RDI better than non-supplement takers [2].

The finding that smoking status was associated with supplement use, is consistent with previous research [1,2,5]. The NHANES 1999–2000 found that former smokers and people who have never smoked were more likely to be supplement users than current smokers [1,5]. The NHANES 1999–2000 reported that 61% of former smokers were dietary supplement users, while 52% of people who had never smoked reported using dietary supplements and only 43% of current smokers reported using dietary supplements [1].

Previous research had mixed findings with regard to alcohol consumption and its association with dietary supplement use [1,5,9,35]. Many studies found that people who consume less alcohol were more likely to take supplements [1,5,35]; however, a study from Canada did not find an association between alcohol consumption and dietary supplement use [9]. The current study did not find an association between alcohol consumption and supplement use. These discrepancies may be due to the type of alcohol consumed, as previous studies have found positive associations for wine consumption, but no associations for beer consumption [1,5,38]. Dietary supplement users are more likely to be those who are more health conscious and therefore adopt health behaviours, such as not smoking and consuming less alcohol, which may motivate them to take dietary supplements [39].

Supplement users are also more likely to have a better health status. Previous research has reported varied conclusions in relation to BMI and its relationship to supplement use; however, the current study did not find an association between BMI and supplement use and found an inverse association between waist circumference and supplement use. A study on supplement use in Taiwan, which focused on the use of multivitamin and mineral supplements, calcium, vitamin E and fish oil, found no association between BMI and supplement use [40]. Similarly, NHANES 2007–2008 did not find an association between supplement use and BMI [10]. On the contrary, a study conducted in the United States on herbal supplements found that herbal and specialty supplement users had lower BMIs [37]. Most studies focus on BMI as an indicator of good health status, with regard to supplement use; however, waist circumference has been regarded as a more accurate indicator of metabolic index, with this measure being used when defining metabolic syndrome [41]. The results of a Danish study showed that people who scored lower in the health index—meaning they had lower blood pressure, a lower waist circumference and did not test positive to the urine glucose test—had higher supplement use [2,5]. In line with our findings, a study conducted in the United Kingdom found that people who had a history of high blood pressure were less likely to be taking dietary supplements [2]. Previous studies have reported an association between self-reported health and supplement use; however, the present study did not find this association [2]. The discrepancy may be due to variations in the populations under study, as our study included a wide age range of participants [42].

Supplement users have been shown to have dietary intakes that tend to be healthier, and higher in a range of nutrients, when considering food intake alone, compared to supplement non-users. Supplement users were reported to have a higher fibre consumption from food only compared to supplement non-users, which is consistent with previous research [43,44]. This is reflective of many nutrients, as the diets of those using dietary supplements are higher in nutrients than non-supplement users. Not surprisingly, when the contribution from supplements is taken into account, supplement users have higher vitamin and mineral intakes, compared to supplement non-users. Previous research has suggested that many people are reaching the upper limit for vitamins and minerals, such as niacin, folate, iron and zinc [11]. In the current study, some nutrients intakes may actually be exceeding the RDI—for example vitamin E, vitamin B12 and zinc—which may result in toxicity [9,45]. It is unclear from the current data how long participants had been consuming the reported supplements, and therefore whether these intakes are higher than the RDI is of concern. Further research focusing on biomarkers of nutritional status may provide insight into understanding the impact of these levels of supplementation.

The strengths of the present study include the nationally-representative data, which make our findings generalizable to the wider population. A limitation to the current study is its cross-sectional design, which did not allow for an investigation of any causal relationships. A further limitation is the differing definitions of supplement users across studies [1,4,5,9,31]. The lack of standardization, in regard to the definition of supplement users, makes it difficult to compare the prevalence between populations. However, despite the methodological differences, similar results, with regard to the demographics, lifestyle habits and health status of supplement users, were identified in a number of studies. Given the widespread use of supplements, further investigation on the social, psychological and economic determinants that motivate the use of supplements is required, to ensure the appropriate use of supplements and to minimise the potential harm which can be caused through excessive use of dietary supplements [32].

## 5. Conclusions

This study examined supplement use within the Australian population, and found that supplement use was higher among females, older adults, those with higher education levels and among those from areas with the least socioeconomic disadvantage. Supplement users were also more likely to participate in healthier lifestyle behaviours, have underlying diets that were higher in many nutrients and have a more favourable health status, when compared to supplement non-users. Future studies examining the potential beneficial or adverse health effects associated with dietary supplements should adjust for other health behaviors and socio-demographic factors, as these variables may confound the associations attributed to dietary supplement use. Future research should focus on understanding intakes of supplements, including understanding long term intakes and whether they are above recommended levels and understanding the drivers of supplement use, to ensure that there is appropriate use of supplements among Australia adults in the community.

## Figures and Tables

**Table 1:** Demographic, lifestyle and health status characteristics of Australian adults in the Australian Health Survey (*n* = 4895).

Characteristic	All Subjects	Supplement Non-Users	Supplement Users	*p*-Value ^1,2^
Sex *n*, (%)				<0.001
Male	2340 (47.8)	1547 (66.1)	795 (34.0)	
Female	2555 (52.2)	1365 (53.3)	1195 (46.7)	
Age, years, *n*, (%)				<0.001
19–30	792 (16.2)	576 (72.7)	216 (27.3)	
31–50	1815 (37.1)	1132 (62.4)	683 (37.6)	
51–70	1648 (33.7)	898 (54.5)	750 (45.5)	
71–85	640 (13.1)	303 (47.3)	337 (52.7)	
Education *n*, (%)				0.006
Secondary school or less	1813 (37.0)	1146 (63.2)	667 (36.8)	
Certificate or diploma	1714 (35.0)	1022 (59.6)	692 (40.4)	
Bachelor degree	911 (18.6)	520 (57.1)	391 (42.9)	
Postgraduate degree	457 (9.3)	221 (48.4)	236 (51.6)	
Area level disadvantage *n*, (%)				<0.001
Most disadvantaged (Lowest 20%)	885 (18.1)	569 (64.3)	316 (35.7)	
Second quintile	999 (20.4)	629 (63.0)	370 (37.0)	
Third quintile	941 (19.2)	539 (57.3)	402 (42.7)	
Fourth quintile	889 (18.2)	505 (56.8)	384 (43.2)	
Least disadvantaged (Highest 20%)	1181 (24.1)	667 (56.5)	514 (43.5)	
Physical activity *n*, (%)				0.005
Met recommended guidelines ^3^	2169 (44.3)	1237 (57.0)	932 (43.0)	
Did not meet recommended guidelines	2726 (55.7)	1672 (61.3)	1054 (38.7)	
Sedentary behavior, min ^4^	3.31 ± 0.29	3.31 ± 0.29	3.30 ± 0.29	0.889
Smoking *n*, (%)				<0.001
Current smoker	844 (17.2)	614 (72.8)	230 (27.2)	
Ex-smoker	1663 (34.0)	914 (55.0)	749 (45.0)	
Never smoked	2388 (48.8)	1381 (57.8)	1007 (42.2)	
Alcohol *n*, (%)				0.691
No alcohol consumed	2570 (52.5)	1542 (60.0)	1028 (40.0)	
One standard drink or less ^5^	2234 (45.6)	1303 (58.3)	931 (41.7)	
More than one standard drink	91 (1.9)	64 (70.3)	27 (29.7)	
Fruit and vegetables *n*, (%)				0.007
Met guidelines ^6^	281 (5.7)	132 (47.0)	149 (53.0)	
Did not meet guidelines	4614 (94.3)	2777 (60.2)	1837 (39.8)	
DGI ^7^ *n*, (%)				<0.001
Low DGI score	2448 (50.0)	1591 (65.0)	857 (35.0)	
High DGI score	2447 (50.0)	1318 (53.9)	1129 (46.1)	
Self-reported health *n*, (%)				0.170
Poor/Fair	780 (15.9)	467 (59.9)	313 (40.1)	
Good	1549 (31.6)	920 (59.4)	629 (40.6)	
Very good	1770 (36.2)	1038 (58.6)	732 (41.4)	
Excellent	796 (16.3)	484 (60.8)	312 (39.2)	
Blood pressure *n*, (%)				0.435
Non-hypertensive	3757 (76.8)	2232 (59.4)	1525 (40.6)	
Hypertensive	1138 (23.3)	677 (59.5)	461 (40.5)	
Chronic disease *n*, (%)				0.377
Absent	4506 (92.1)	2688 (59.7)	1818 (40.4)	
Present	389 (8.0)	221 (56.8)	168 (43.2)	
Waist circumference, cm				0.472
No risk	1611 (32.9)	976 (60.6)	635 (39.4)	
Increased risk	1156 (23.6)	691 (59.8)	465 (40.2)	
Substantially increased risk	2128 (43.5)	1242 (58.4)	886 (41.6)	
BMI ^8^, kg/m^2^				0.186
Underweight	65 (1.3)	42 (64.6)	23 (35.4)	
Normal	1642 (33.5)	958 (58.3)	684 (41.7)	
Overweight	1820 (37.2)	1069 (58.7)	751 (41.3)	
Obese	1368 (28.0)	840 (61.4)	528 (38.6)	

^1^ *p*-Values comparing supplement users to non-supplement users, determined using chi square for categorical variables; ^2^ *p*-Values comparing supplement users to non-supplement users, determined using *t*-tests for continuous variables; ^3^ One hundred and fifty min and five sessions a week; ^4^ Values represent mean and standard error (SE); ^5^ A standard drink is defined as 10 grams of alcohol; ^6^ Five serves of vegetables and two serves of fruit per day; ^7^ Dietary guideline index; ^8^ Body mass index (BMI; kg/m^2^) is calculated as weight (kg) divided by height (m^2^).

**Table 2:** Dietary supplement use (prevalence ratio and 95% confidence intervals) across demographic and lifestyle characteristics in adults from the Australian Health Survey (*n* = 4895).

Characteristic	Crude		Adjusted ^1^	
	Supplement User ^2^	*p-*Value ^3^	Supplement User ^2^	*p-*Value ^4^
Sex		<0.001		<0.001
Male (reference)	1.00		1.00	
Female	1.26 (1.12, 1.42)		1.24 (1.10, 1.39)	
Age group (years)		<0.001		<0.001
19–30 (reference)	1.00		1.00	
31–50	1.32 (1.06, 1.64)		1.31 (1.05, 1.64)	
51–70	1.55 (1.24, 1.94)		1.54 (1.23, 1.93)	
71–85	1.87 (1.48, 2.36)		1.83 (1.45, 2.31)	
Education		0.006		<0.001
Secondary school or less (reference)	1.00		1.00	
Certificate or diploma	1.16 (0.99, 1.36)		1.25 (1.07, 1.46)	
Bachelor degree	1.28 (1.07, 1.53)		1.43 (1.20, 1.71)	
Postgraduate degree	1.40 (1.14, 1.72)		1.53 (1.25, 1.88)	
Area level disadvantage		<0.001		<0.001
Lowest 20% (reference)	1.00		1.00	
Second quintile	1.18 (0.98, 1.41)		1.22 (1.02, 1.46)	
Third quintile	1.39 (1.13, 1.71)		1.44 (1.20, 1.75)	
Fourth quintile	1.44 (1.20, 1.73)		1.52 (1.28, 1.80)	
Highest 20%	1.49 (1.25, 1.77)		1.57 (1.33, 1.86)	
Physical activity		0.005		<0.001
Met recommended guidelines (reference)	1.00		1.00	
Did not meet recommended guidelines	0.86 (0.77, 0.95)		0.79 (0.71, 0.88)	
Sedentary behaviour (min/day)	0.99 (0.80, 1.21)	0.889	1.12 (0.89, 1.39)	0.330
Smoking		<0.001		<0.001
Current smoker (reference)	1.00		1.00	
Ex-smoker	1.70 (1.43, 2.03)		1.56 (1.31, 1.86)	
Never smoked	1.52 (1.31, 1.77)		1.46 (1.25, 1.70)	
Alcohol		0.676		0.875
No alcohol consumed (reference)	1.00		1.00	
One standard drink or less	1.34 (0.93, 1.16)		1.02 (0.92, 1.14)	
More than one standard drink	0.91 (0.52, 1.58)		0.98 (0.56, 1.72)	
Fruit and vegetables		0.004		0.024
Met guidelines (reference)	1.00		1.00	
Did not meet guidelines	0.75 (0.63, 0.91)		0.81 (0.68, 0.97)	
DGI		<0.001		<0.001
Low DGI score (reference)	1.00		1.00	
High DGI score	1.38 (1.26, 1.52)		1.32 (1.20, 1.44)	
Self-assessed health		0.265		0.053
Poor/Fair (reference)	1.00		1.00	
Good	1.07 (0.91, 1.27)		1.18 (0.99, 1.40)	
Very good	1.13 (0.97, 1.32)		1.26 (1.07, 1.49)	
Excellent	0.96 (0.79, 1.17)		1.10 (0.90, 1.34)	

^1^ Poisson regression model adjusted for sex and age; ^2^ Prevalence ratio (95% CI); ^3^ *F*-test was conducted to obtain an overall *p*-value for categorical variables; ^4^ *F*-test was conducted to obtain an overall *p*-value for categorical variables.

**Table 3:** Dietary supplement use (prevalence ratio and 95% confidence intervals) across health-related characteristics in adults from the Australian Health Survey (*n* = 4895).

Characteristic	Crude		Adjusted ^1^	
	Supplement User ^2^	*p-*Value ^3^	Supplement User ^2^	*p-*Value ^4^
Blood pressure		0.443		0.031
Non-hypertensive (reference)	1.00		1.00	
Hypertensive	0.95 (0.85, 1.08)		0.87 (0.77, 0.99)	
Chronic disease		0.372		0.734
Absent (reference)	1.00		1.00	
Present	1.10 (0.89, 1.35)		0.97 (0.79, 1.18)	
Waist Circumference (cm)		0.411		0.007
No risk (reference)	1.00		1.00	
Increased risk	0.97 (0.85, 1.21)		0.89 (0.79, 1.01)	
Substantially increased risk	0.94 (0.84, 1.04)		0.83 (0.75, 0.93)	
BMI (kg/m^2^)		0.120		0.081
Underweight (reference)	1.00		1.00	
Normal	1.11 (0.65, 1.89)		0.89 (0.51, 1.55)	
Overweight	1.10 (0.62, 1.96)		0.86 (0.48, 1.54)	
Obese	0.95 (0.58, 1.57)		0.75 (0.45, 1.27)	

^1^ Poisson regression model, adjusted for sex, age, education, area level disadvantage, smoking, fruit and vegetable consumption and DGI; ^2^ Prevalence ratio (95% CI); ^3^ *F*-test was conducted to obtain an overall *p*-value for categorical variables; ^4^ *F*-test was conducted to obtain an overall *p*-value for categorical variables.

**Table 4:** Type of dietary supplements used by age and sex.

	Total	Age				Sex			
	*n*, (%) ^1^	19–30 *n*, (%)	31–50 *n*, (%)	51–70 *n*, (%)	71–85 *n*, (%)	*p*-Value ^2^	Males *n*, (%)	Females *n*, (%)	*p*-Value ^3^
Multivitamin ^4^	1031 (15.2)	133 (12.9)	426 (41.3)	342 (33.2)	130 (12.6)	0.051	395 (38.3)	636 (61.7)	**<0.001**
Mineral ^5^	739 (10.9)	65 (8.8)	221 (29.9)	318 (43.0)	135 (18.3)	**<0.001**	246 (33.3)	493 (66.7)	**<0.001**
Vitamins ^6^	1230 (18.1)	166 (13.5)	498 (40.5)	413 (33.6)	153 (12.4)	0.693	498 (40.5)	732 (59.5)	**0.004**
Lipid ^7^	998 (14.7)	81 (8.1)	308 (30.9)	436 (43.7)	173 (17.3)	**<0.001**	407 (40.8)	591 (59.2)	**0.019**
Herbal ^8^	1063 (15.6)	112 (10.5)	367 (34.5)	421 (39.6)	163 (15.3)	**0.002**	393 (37.0)	670 (63.0)	**<0.001**
Iron	802 (11.8)	118 (14.7)	341 (42.5)	248 (30.9)	95 (11.9)	0.497	309 (38.5)	493 (61.5)	**0.002**
Folic acid	932 (13.7)	129 (13.8)	398 (42.7)	297 (31.9)	108 (11.6)	0.181	380 (40.8)	552 (59.2)	**0.009**

^1^ Respondents may have reported using more than one supplement; ^2^ *p*-Value relates to comparison of age categories and different supplement types, determined using chi square; ^3^ *p*-Value relates to comparison of sex and different supplement types, determined using chi square; ^4^ Multivitamin and/or multimineral, multivitamin and/or multimineral, with herbal extracts and multivitamin and/or multimineral containing caffeine; ^5^ Mineral supplements include calcium supplements, magnesium supplements, zinc supplements, iodine supplements, selenium supplements and other single mineral supplements; ^6^ Vitamin supplements include vitamin C supplements, vitamin E supplements, folic acid supplements, vitamin D supplements and other single vitamin supplements; ^7^ Lipid supplements include fish oil supplements, fish oil supplements with added nutrients, fish liver oil supplements, evening primrose oil supplements, long chain omega 3 fatty acid supplements and other lipid supplements; ^8^ Herbal supplements include all herbal supplements including those containing caffeine, homoeopathic supplements, protein or amino acid supplements, probiotic supplements, propolis or other bee product supplements, glucosamine and/or chondroitin based supplements, coenzyme q10 supplements and other supplements.

**Table 5:** Analyses for the association of nutrient intake between dietary supplement users and non-supplement users for adults from the Australian Health Survey (*n* = 4895).

	RDI ^3^	Supplement Non-User	Supplement User			
		Food Only Mean ± SD	Food Only Mean ± SD	Food & Supplements Mean ± SD	*p*-Value ^1,4^	*p*-Value ^2,4^
Energy (kJ)		8489.4 ± 2982.7	8371.5 ± 2842.8	8371.5 ± 2842.8	0.014	0.014
Fat (g)		72.0 ± 32.2	71.0 ± 31.4	71.9 ± 31.5	0.607	0.660
Protein (g)	46–81	89.6 ± 34.2	89.8 ± 33.2	89.9 ± 33.3	0.045	0.035
Carbohydrates (g)		219.6 ± 87.9	215.1 ± 83.7	215.1 ± 83.7	0.802	0.802
Fibre (g)	25–30	21.9 ± 9.7	24.5 ± 10.7	24.5 ± 10.7	<0.001	<0.001
Calcium (mg)	1000–1300	779.0 ± 385.9	817.3 ± 359.8	940.5 ± 425.5	<0.001	<0.001
Magnesium (mg)	310–420	326.0 ± 125.2	352.4 ± 129.1	398.2 ± 163.9	<0.001	<0.001
Iron (mg)	8–18	10.8 ± 4.7	11.3 ± 4.6	13.5 ± 8.0	<0.001	<0.001
Zinc (mg)	8–14	10.8 ± 4.8	11.0 ± 4.8	15.7 ± 9.8	0.107	<0.001
Iodine		169.9 ± 72.8	167.9 ± 72.1	204.5 ± 115.6	0.645	<0.001
Selenium		88.0 ± 42.9	91.7 ± 52.7	99.7 ± 55.5	<0.001	<0.001
Vitamin C (mg)	45	95.4 ± 81.1	111.2 ± 85.4	269.8 ± 598.1	<0.001	<0.001
Vitamin E (mg)	7–10	9.8 ± 5.2	10.9 ± 6.1	28.5 ± 65.6	<0.001	<0.001
Folate (natural) (µg)	400	276.2 ± 124.1	309.7 ± 131.6	309.7 ± 131.6	<0.001	<0.001
Folic acid (µg)		197.9 ± 142.9	173.9 ± 137.8	292.6 ± 226.4	0.001	<0.001
B12 (µg)	2.4	4.5 ± 3.7	4.6 ± 3.3	31.4 ± 114.9	0.252	<0.001
Omega-3		259.7 ± 463.3	304.6 ± 574.0	608.2 ± 869.5	0.065	<0.001

^1^ *p*-Value relates to a comparison of supplement non-users’ and supplement users’ food only intakes, determined using an independent *t*-test; ^2^ *p*-Value relates to comparison of supplement non-users’ and supplement users’ total nutrient intakes, determined using an independent *t*-test; ^3^ Recommended daily intake for adults, according to the National Health and Medical Research Council [29]; ^4^ Log transformation, performed for total zinc, food only vitamin C, total vitamin C, total vitamin E, food only folic acid, total folic acid, food only B12, total B12, food only long chain omega 3 fatty acids and total long chain omega 3 fatty acids.

## Abbreviations

SD	standard deviation
NNPAS	National Nutrition and Physical Activity Survey
BMI	body mass index
NHANES	National Health and Nutrition Examination Survey
PR	Prevalence ratios
